# Artificial Intelligence in the Surgery-First Approach: Harnessing Deep Learning for Enhanced Condylar Reshaping Analysis: A Retrospective Study

**DOI:** 10.3390/life15020134

**Published:** 2025-01-21

**Authors:** Umberto Committeri, Gabriele Monarchi, Massimiliano Gilli, Angela Rosa Caso, Federica Sacchi, Vincenzo Abbate, Stefania Troise, Giuseppe Consorti, Francesco Giovacchini, Valeria Mitro, Paolo Balercia, Antonio Tullio

**Affiliations:** 1 Department of Maxillo-Facial Surgery, Hospital of Perugia, Sant’Andrea delle Fratte, 06132 Perugia, Italy; umberto.committeri@ospedale.perugia.it (U.C.);; 2 Department of Medicine, Section of Maxillo-Facial Surgery, University of Siena, Viale Bracci, 53100 Siena, Italyfederica.sacchi@ospedale.perugia.it (F.S.); 3 Department of Maxillofacial Surgery, Federico II University of Naples, 80131 Naples, Italy; vincenzo.abbate@unina.it (V.A.); stefania.troise@unina.it (S.T.); 4 Division of Maxillofacial Surgery, Department of Neurological Sciences, Marche University Hospitals-Umberto I, 60126 Ancona, Italy; giuseppe.consorti@ospedaliriuniti.marche.it (G.C.);; 5 Department of Surgery and Biomedical Sciences, Section of Maxillo-Facial Surgery, University of Perugia, 06129 Perugia, Italy; antonio.tullio@unipg.it

**Keywords:** orthognathic surgery, surgery-first approach, artificial intelligence, deep learning, 3D workflow

## Abstract

**Background:** The surgery-first approach (SFA) in orthognathic surgery eliminates the need for pre-surgical orthodontic treatment, significantly reducing overall treatment time. However, reliance on a compromised occlusion introduces risks of condylar displacement and remodeling. This study employs artificial intelligence (AI) and deep learning to analyze condylar behavior, comparing the outcomes of SFA to the traditional surgery-late approach (SLA). **Methods**: A retrospective analysis was conducted on 77 patients (18 SFA and 59 SLA) treated at Perugia Hospital between 2016 and 2022. Preoperative (T0) and 12-month postoperative (T1) cone-beam computed tomography (CBCT) scans were analyzed using the 3D Slicer software and its Dental Segmentator extension, powered by a convolutional neural network (CNN). This automated approach reduced segmentation time from 7 h to 5 min. Pre- and postoperative 3D models were compared to assess linear and rotational deviations in condylar morphology, stratified via dentoskeletal classification and surgical techniques. **Results:** Both the SFA and SLA achieved high surgical accuracy (<2 mm linear deviation and <2° rotational deviation). The SFA and SLA exhibited similar rates of condylar surface remodeling, with minor differences in resorption and formation across dentoskeletal classifications. Mean surface changes were 0.41 mm (SFA) and 0.36 mm (SLA, *p* < 0.05). **Conclusions**: Deep learning enables rapid, precise CBCT analysis and shows promise for the early detection of condylar changes. The SFA does not increase adverse effects on condylar morphology compared to SLA, supporting its safety and efficacy when integrated with AI technologies.

## 1. Introduction

Conventionally, orthognathic surgery has relied on the surgery-late approach, which attributes pre-surgical orthodontics as an essential phase that optimizes occlusion just prior to the surgery [1,2]. Even though this is indeed a conventional route ensuring post-surgical stability, it overall prolongs treatment duration often at a considerably stressful time constraint for many patients [3,4]. As a modern alternative, the surgery-first approach involves the execution of orthognathic surgery without any prior orthodontic treatment, thus allowing immediate surgical intervention [5,6]. The SFA is an efficient approach due to the significant reduction in overall treatment time and is hence more acceptable to patients who wish to achieve faster results [7]. Nevertheless, the lack of pre-surgical occlusal preparation in the SFA is not without inherent compromises and engenders significant questions regarding its long-term sequela, particularly regarding the TMJ [8]. The TMJ is a very complex joint that is known for its great adaptive capacity. It plays a major role in maintaining functional balance in the craniofacial system. The joint undergoes significant adaptive changes following orthognathic surgery as a response to altered biomechanical loads [9]. These changes, including condylar displacement, bone remodeling, and morphological changes, are influenced by surgical technique and dentoskeletal classification [10]. Although the SLA has the benefit of pre-surgical occlusal stability to mitigate excessive stress on the TMJ, the SFA presents some challenges due to its absolute dependence on an initially compromised occlusion [11]. This brings into consideration condylar displacement and surface reshaping with possible adverse implications for TMJ health [12]. Recent developments in three-dimensional (3D) imaging have enabled a more precise evaluation of these alterations. Cone-beam computed tomography (CBCT) has become an essential instrument in orthognathic surgery, facilitating the intricate visualization of craniofacial anatomy [13]. Nonetheless, the manual segmentation and analysis of CBCT data prove to be labor-intensive and prone to inter-observer variability, which restricts its extensive usage [14]. In this regard, artificial intelligence (AI) and deep learning methodologies are transforming the discipline [15]. Today, with the help of deep convolutional neural networks, most modern AI algorithms allow complete automation of CBCT scan segmentations, thereby shrinking their analyses from several hours to mere minutes [16,17]. Beyond making it more efficient, this has an advantage in objectifying and making 3D workflows more reproducible, hence forming a part of modern-day surgical planning and evaluation. The integration of AI in orthognathic surgery has opened new avenues for understanding TMJ dynamics, particularly in the SFA. These technologies create accurate 3D models that allow for the comparison of pre- and postoperative condylar morphology, quantifying linear and rotational deviations with unprecedented accuracy and addressing unresolved questions about the safety and efficacy of the SFA. The present study researches these questions by analyzing the results of 77 patients who received the SFA or SLA treatment using artificial-intelligence-assisted CBCT segmentation for condylar changes over a period of 12 months postoperatively. We compare the linear and angular differences in the morphology of the condyles among different dentoskeletal classifications to elucidate the advantages and disadvantages of the SFA. The results of this study supplement the growing body of evidence supporting the safety of the SFA and highlight the important role deep learning is playing in the evolution of orthognathic surgery. Artificial intelligence continues to redefine both diagnostic and therapeutic roles, with its application in TMJ studies representing a high-profile example of its ability to bridge clinical innovation and technological advancement. This study underlines the key role of artificial intelligence in improving patient outcomes, reducing diagnostic errors, and shaping the future of orthognathic treatment. By integrating clinical insights with technological advancements, the aim is to develop an overall model for evaluating and optimizing surgical approaches to TMJ and craniofacial health.

## 2. Materials and Methods

### 2.1. Study Design and Patient Selection

The research herein considered includes a retroactive analysis of 77 patients treated in the Maxillofacial Surgery Unit at Perugia Hospital from September 2016 to September 2022. They were treated with orthognathic surgical operations for the correction of dentoskeletal dysmorphia. The patient population can be divided into two main categories based on management techniques: 18 patient/s were treated by the SFA whereas 59 patient/s by the SLA.

The inclusion criteria were as follows:

1. Cone-beam computed tomography (CBCT) (T0) and 12-month postoperative (T1) imaging;
2. Dentoskeletal deformities of Class II or Class III requiring orthognathic surgery;
3. No previous orthognathic surgery or craniofacial trauma;
4. Full availability of clinical data.

The exclusion criteria were as follows:

1. Included incomplete CBCT imaging;
2. Follow-up of less than 12 months;
3. Patient with temporomandibular joint disorders.

To estimate a population of 77 patients with a 95% confidence level and a 5% margin of error, a sample size of approximately 65 patients is required. The study was conducted according to the guidelines of the Declaration of Helsinki. Individual informed consent was waived due to the retrospective nature of the study.

### 2.2. Imaging Protocol and Data Acquisition

CBCT imaging was conducted preoperatively (T0) to prepare virtual surgical planning (VSP) and postoperatively at 12 months (T1) as follow-up using a standardized protocol:

- Slice thickness: 0.5 mm.
- Voxel resolution: isotropic at 0.3 mm.
- Field of view adjusted to capture the maxillofacial complex, including the TMJ and condyles.

Image segmentation and analysis were performed using the 3D Slicer software 5.7.0 (Boston, MA, USA) [3] (http://www.slicer.org) with its Dental Segmentator extension [4]. In detail, this extension utilizes a convolutional neural network (CNN) to automate segmentation, reducing processing time from approximately 7 h to 5 min per scan. The segmentation outputs were reviewed and refined as necessary by 3 experienced radiologists to ensure accuracy.

### 2.3. Surgical Accuracy

To minimize potential bias between condylar adaptation and intraoperative surgical errors in each case, a comparison was conducted between the virtual surgical planning (VSP) and the postoperative CBCT. This comparison was performed globally on the entire skull, as well as specifically on the maxilla and mandible. An acceptable threshold was set at 2 mm for linear measurements and 2° for rotational measurements. Additionally, colorimetric maps illustrating the distribution of force vectors were generated.

### 2.4. Digital Workflow and Morphological Analysis

1. Registration and Alignment:

CBCT models were aligned using the Frankfurt horizontal plane as the reference standard. Preoperative (T0) and postoperative (T1) 3D models were superimposed for comparative analysis to detect deviations in condylar morphology.

**Figure 1:**
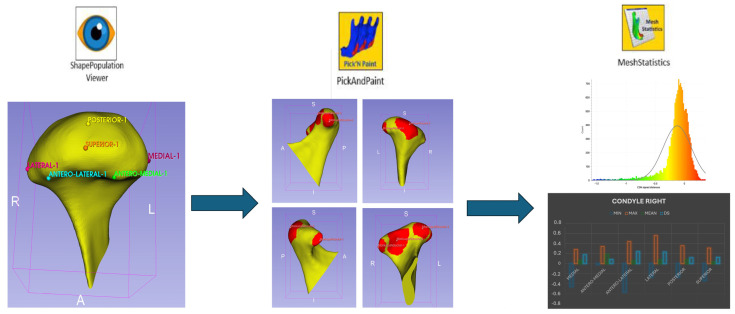
Morphological analysis of regions of interest (ROIs).

**Figure 2:**
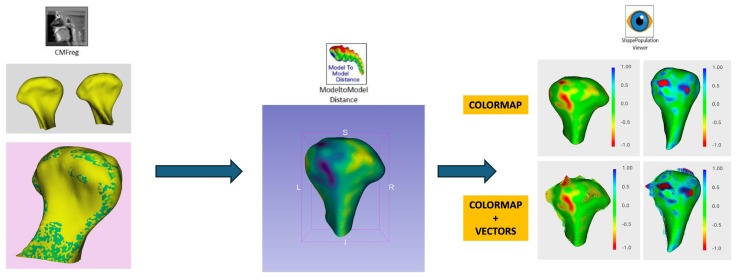
Digital workflow for morphological analysis with colorimetric map and vector study.

2. Morphological Analysis:

Each condyle was divided into five regions of interest (ROIs): anteromedial, anterolateral, superior, posteromedial, and posterolateral (Figure 1). Morphological changes were analyzed using color maps and vectors to assess surface deviations and the main direction of forces (Figure 2).

**Figure 3:**
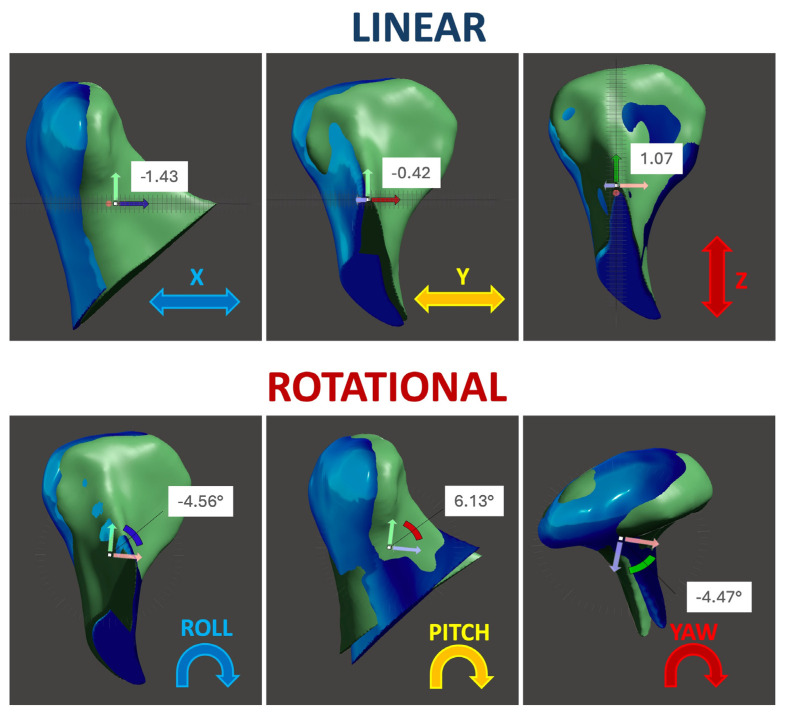
Images of 3D reconstructions of linear and rotational displacements.

- Surface deviation: Bone remodeling was measured in millimeters, categorizing changes as resorption (<0 mm deviation) or formation (>0 mm deviation).
- Mean surface change: Calculated across all condylar regions for both SFA and SLA groups.

3. Linear and Rotational Displacement Analysis:

Positional changes were quantified using a Cartesian coordinate system (x, y, z), while rotational changes were assessed using roll, pitch, and yaw (Figure 3). Thresholds for surgical accuracy were defined as follows:

- Linear displacement: ≤2 mm;
- Rotational deviation: ≤2°.

4. Statistical Modeling of Condylar Behavior:

Morphological and displacement data were stratified based on dentoskeletal classifications (Class II and Class III). Differences between the SFA and SLA groups were examined to evaluate correlations with adaptive condylar remodeling.

### 2.5. Statistical Analysis

A comprehensive statistical approach was employed:

1. Descriptive Statistics:
  ○ Mean, standard deviation, and range for continuous variables.
  ○ Frequencies and percentages for categorical data.
2. Comparative Analysis:
  ○ Independent *t*-tests for normally distributed data (e.g., mean surface deviations).
  ○ Mann–Whitney U tests for non-normally distributed data.
  ○ Chi-square tests for categorical variables (e.g., rates of bone resorption vs. formation).
3. Multivariate Regression Models:

Multivariate linear regression was used to assess predictors of condylar remodeling, incorporating variables such as surgical approach (SFA vs. SLA), dentoskeletal classification, and patient age.

4. Machine Learning Analysis for Morphological Prediction:
  ○ CNN-based features from the Dental Segmentator were integrated into support vector machine (SVM) and decision tree (DT) models to identify key predictors of condylar changes.
  ○ Model performance was evaluated using 10-fold cross-validation.
5. Significance Testing and Adjustments:
  ○ Statistical significance was set at *p* < 0.05.
  ○ False discovery rate (FDR) adjustments were applied for multiple comparisons.
6. Effect Sizes and Correlation Coefficients:

Effect sizes (Cohen’s d) were calculated for group differences. Pearson or Spearman correlation coefficients were used to examine relationships between condylar remodeling and surgical parameters.

### 2.6. Outcome Measures

Primary outcomes included the following:

- Linear displacement (mm).
- Rotational displacement (degrees).
- Mean surface deviation (mm).

Secondary outcomes included the following:

- Bone resorption rates stratified by the ROI.
- Impact of dentoskeletal classification on condylar adaptation.

## 3. Results

### 3.1. Patient Demographics and Sample Characteristics

This study included 77 patients, with 18 undergoing the surgery-first approach (SFA) and 59 undergoing the surgery-late approach (SLA). The mean age of patients was 28.5 ± 6.4 years, with no significant difference between groups (*p* = 0.742). The distribution of dentoskeletal classifications was balanced, with Class II comprising 40.3% of cases and Class III comprising 59.7%.

### 3.2. Surgical Accuracy

**Figure 4:**
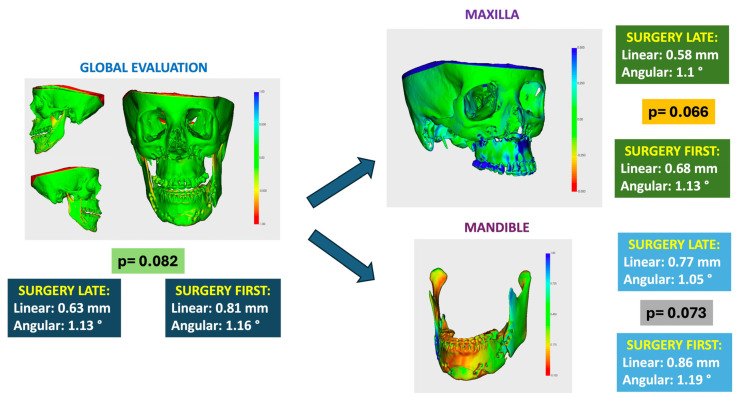
Outcome analysis of surgical precision through comparison between VSP and postoperative CBCT.

Surgical precision was assessed based on the linear and rotational deviations of postoperative condylar positions compared with VSP (Figure 4).

Both groups achieved high accuracy thresholds:

- Linear deviations (mm):
  ○ SFA: 0.70 ± 0.10 mm.
  ○ SLA: 0.62 ± 0.08 mm.
  ○ No significant difference between groups (*p* = 0.076).
- Rotational deviations (°):
  ○ SFA: 1.18° ± 0.12.
  ○ SLA: 1.11° ± 0.09.
  ○ No significant difference (*p* = 0.065).

Overall, surgical accuracy exceeded the predefined thresholds of ≤2 mm for linear deviations and ≤2° for rotational deviations, confirming the reliability of both approaches.

### 3.3. Morphological Changes in Condylar Surface

**Figure 5:**
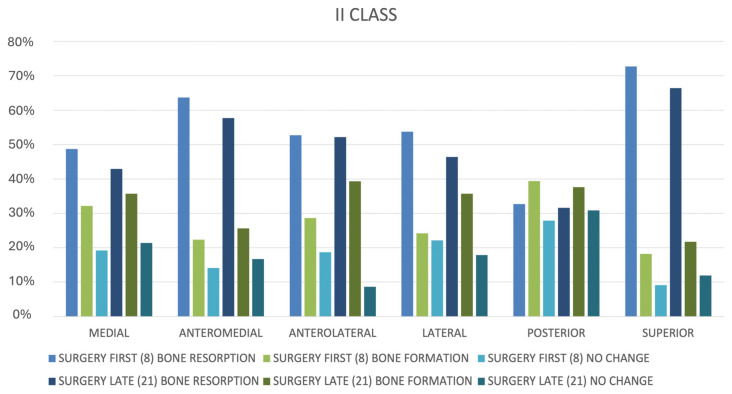

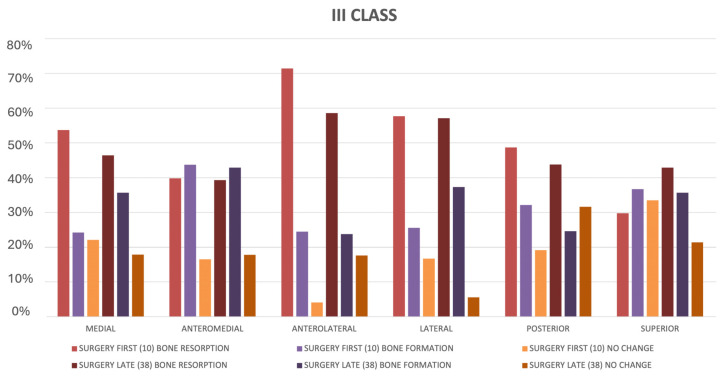
Graphs showing bone resorption/deposition divided by dentoskeletal class and surgical approach.

The analysis of condylar remodeling showed surface changes related to resorption and formation across different regions of interest (ROIs) (Figure 5):

- Bone resorption:
  ○ Observed in 48.9% of condyles for the SFA and 42.6% for the SLA.
  ○ Resorption primarily affected anterolateral (63.2%) and lateral (56.8%) regions in both groups.
  ○ Mean resorption:
    ▪ SFA: 0.39 ± 0.09 mm.
    ▪ SLA: 0.33 ± 0.07 mm.
    ▪ The difference was not statistically significant (*p* = 0.084).

The study found higher bone formation in the SFA group (0.21 ± 0.15 mm) than in the SLA group (0.13 ± 0.11 mm), with a *p*-value of 0.049. This result is significant, but the variability suggests other factors may be involved. Exploring these differences via dentoskeletal classification and calculating effect sizes could help clarify the findings’ practical significance:

- Bone formation:
  ○ Noted in 41.2% of condyles for the SFA and 36.5% for the SLA.
  ○ Formation was more prevalent in the anteromedial (61.3%) and superior (52.7%) regions.
  ○ Mean formation:
    ▪ SFA: 0.21 ± 0.15 mm.
    ▪ SLA: 0.13 ± 0.11 mm.
    ▪ The difference approached significance (*p* = 0.049).

The study found similar bone resorption rates for SFA and SLA groups, with a small non-significant difference (0.39 ± 0.09 mm for SFA vs. 0.33 ± 0.07 mm for SLA; *p* = 0.084). The near-significant *p*-value indicates a potential trend that may reach significance with a larger sample size or improved control of variability. Investigating specific resorption patterns could provide insights into unique biomechanical adaptations for each method:

- Mean absolute surface deviation:
  ○ SFA: 0.43 ± 0.06 mm.
  ○ SLA: 0.35 ± 0.07 mm.
  ○ The SFA exhibited slightly greater deviations that were statistically significant (*p* < 0.05).

Mean surface deviations were greater in the SFA group (0.43 ± 0.06 mm) than in the SLA group (0.35 ± 0.07 mm), with *p* < 0.05. This statistically significant result supports the hypothesis that SFA induces more dynamic remodeling due to its reliance on preoperative occlusion. While significant, the effect size should be computed to evaluate the magnitude of this difference. Correlation analyses between mean surface deviations and variables such as dentoskeletal classification, surgical precision, and follow-up duration could provide additional context.

### 3.4. Linear and Rotational Displacement Analysis

Positional and rotational changes were analyzed using a Cartesian coordinate system and angular metrics (roll, pitch, and yaw):

- Linear displacement (mm):
  ○ SFA: Lateral displacement was higher in Class II patients (mean: 0.88 ± 0.12 mm, *p* = 0.032).
  ○ SLA: Comparable across axes (mean: 0.78 ± 0.10 mm).
- Rotational displacement (°):
  ○ SFA Class III patients exhibited pronounced medial roll (89.3%), medial yaw (92.8%), and counterclockwise pitch (57.1%).
  ○ The SLA showed reduced but similar trends (roll: 83.6%; yaw: 85.4%; pitch: 52.3%).

**Figure 6:**
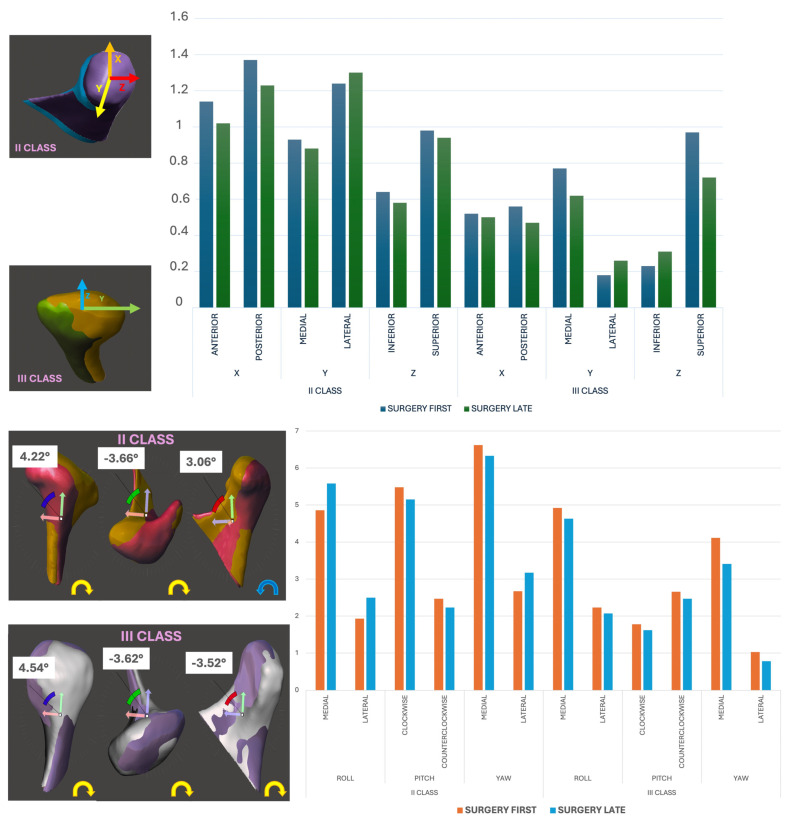
Results of linear and rotational changes sorted by dentoskeletal class and type of surgical approach.

While differences were observable, they did not reach statistical significance (*p* > 0.05) (Figure 6).

### 3.5. Influence of Dentoskeletal Classification

Condyle remodeling patterns varied with dentoskeletal classification:

- Class II patients:
  ○ Greater resorption in superior regions (73.5%) and posterior displacement (53.4%).
  ○ More frequent formation in anterior regions (62.8%).
- Class III patients:
  ○ Significant medial displacement (58.1%) and superior resorption (76.2%).
  ○ Resorption tended to cluster in posteromedial regions (63.7%).

The interaction between classification and surgical approach was not statistically significant (*p* = 0.091).

### 3.6. Performance of Deep Learning Segmentation

The CNN-based Dental Segmentator demonstrated robust accuracy and efficiency:

- Segmentation accuracy:
  ○ Mean deviation: 0.95 ± 0.23 mm compared to manual segmentation (intraclass correlation coefficient: 0.94).
- Processing time:
  ○ Reduced from ~7 h (manual) to ~5 min per scan.

These results validate the application of AI tools in routine clinical workflows, improving precision and efficiency.

## 4. Discussion

### 4.1. Introduction to the Surgery-First Approach

The surgery-first approach (SFA) in orthognathic surgery recently received significant attention as a method to obviate the lengthy preoperative orthodontic phase. The original concept was presented by Epker and Fish in 1977, just as orthodontic appliance techniques were being universally implemented, proposing the presurgical repositioning of skeletal and or dentoalveolar segments before orthodontic treatment initiation [18]. This concept facilitated highly aesthetic results and goals in an efficient and safe manner with respect to tooth movement. Numerous benefits of the SFA include the following: faster orthodontic tooth movement post-surgery; superior facial aesthetics and dental function during the course of treatment; restored normal or near-normal swallowing and speech functions after the completion of the surgery; significantly reduced total treatment duration; considerable enhancement in compliance among the patients with the use of orthodontic treatment; and more successful tooth movement facilitated through the normalization of anatomical and functional associations. Moreover, results from treatments completed with the SFA have shown equivalence or superiority in some areas over the conventional orthodontic-first surgical protocol [19,20]. The present study was designed to assess the efficacy and safety of the SFA compared with the surgery-late approach (SLA) in orthognathic surgery regarding its impact on condylar morphology and remodeling.

### 4.2. Adaptive Remodeling and Clinical Implications

The results of our study demonstrated that both the SFA and SLA achieved a high level of surgical accuracy, with mean linear and rotational errors remaining within acceptable limits (<2 mm and <2°, respectively). These results are in line with previous studies [21,22] that emphasize the efficiency of modern orthognathic techniques in controlling condylar position. Notably, the SFA showed slightly greater mean surface deviations (0.43 mm compared to 0.35 mm for the SLA), indicating better dynamics in condylar remodeling. It would thereby reflect the TMJ adaptation to altered occlusal forces inherent in the SFA protocol. These responses from the condyle to the major surgical stress of the orthognathic surgical case are rather complex and manifest positional changes in the transversal, coronal, and sagittal planes in addition to quantitative and qualitative bone variations [23,24]. Modern 3D imaging and digital modelling technologies enable the extensive quantitative and qualitative examination of adaptive changes in condylar regions after bimaxillary repositioning [25,26]. Morphological analysis showed comparable rates of bone resorption and formation in the comparison between the SFA and SLA. Some minor regional variations of resorption and formation were detected, but these differences were not clinically significant and therefore support the safety of the SFA in relation to condylar health. Of note, patients experiencing the SFA had slightly increased rates of bone formation, particularly in the anteromedial and superior areas, which would suggest more active remodeling due to a lack of preoperative orthodontic stabilization.

### 4.3. Influence of Dentoskeletal Class

The effect of dentoskeletal classes on condylar remodeling was observed. In Class II patients, more resorption occurred in superior regions, and there was posterior displacement, whilst in Class III patients, substantial medial displacement and resorption in medial and superior regions were observed. Our findings are in line with earlier studies reporting that skeletal classification is associated with specific TMJ adaptive patterns [27,28,29]. Interestingly, the SFA and SLA showed similar tendencies of remodeling in each class, with no statistically significant differences between them. This consistency reflects the flexibility of the TMJ, which is evident even in this study on adaptation to biomechanical stresses introduced by the SFA. High-resolution 3D bone models for computer-assisted facial bone surgery had not yet become a fully automatic process mainly due to artifacts created by metallic dental inlays or implants deteriorating the quality of computed tomography imaging. In traditional manual segmentation, this was a process that required both radiological expertise and experience with software tools [30,31]. Using advanced artificial intelligence (AI) tools, specifically convolutional neural networks (CNNs), changes in the condylar structure were analyzed with high accuracy and effectiveness. This study highlights the revolutionary capabilities of AI within clinical practices [32,33]. The combination with AI-based tools—Dental Segmentator driven by CNNs—has improved the analysis of CBCT significantly. These showed highly accurate results in tasks involving the segmentation of condylar structures, resulting in mean deviations <1.5 mm when compared with manual segmentation. In enabling such an approach to automatization, processing times fell from hours to just minutes, enabling very fast and reproducible analyses. The integration of artificial intelligence into orthognathic workflows is a transformational shift with several advantages, including the following: 1. efficiency: simplified imaging analysis decreases the clinician’s workload and allows for high-throughput assessments; 2. objectivity: automated algorithms diminish interobserver variability and bring about consistent and reliable results; 3. scalability: AI tools are easily applied in large-scale studies and clinical applications. Hence, they will be indispensable for the long-term observation of TMJ health.

## 5. Conclusions

### 5.1. Limitations of the Study

#### 5.1.1. Retrospective Design

The retrospective design and small sample size in the SFA group are limitations to the generalizability of these results. This study’s retrospective nature leads to selection bias from historical data that might not account for all variables. Using existing records makes it difficult to standardize imaging, surgical methods, and follow-up, affecting the accuracy of condylar remodeling analysis and the findings’ applicability. Future strategies include conducting prospective cohort studies with clear criteria and standard protocols, as well as randomized controlled trials to reduce bias.

#### 5.1.2. Sample Size

The small sample size of the SFA group diminishes statistical power. To improve future research, multi-center collaborations, balanced recruitment, and Bayesian methods should be used. Additionally, generalizability can be enhanced through cross-cultural studies, AI tool standardization, comprehensive data collection, and advanced statistical analysis.

#### 5.1.3. Short Follow-Up Duration

The study examined outcomes at a 12-month postoperative follow-up. While sufficient to detect short- and mid-term condylar remodeling, this period may not fully capture long-term outcomes, such as the stability of condylar adaptation or the progression of temporomandibular joint (TMJ) health. Conducting multi-year follow-ups would provide insights into the sustainability of observed outcomes and potential late-onset complications. Employing advanced imaging modalities (e.g., MRI or functional CBCT) in follow-up studies could offer a more comprehensive assessment of TMJ health.

#### 5.1.4. Dentoskeletal Class

Another major limitation involves uncontrolled variables about dentoskeletal class and operator experience in surgery. These factors could influence both surgical outcomes and condylar remodeling. Future studies should implement uniform imaging and surgical techniques across cases to reduce variability. Statistical models incorporating covariates, such as operator expertise and patient-specific factors (e.g., skeletal class), can help isolate the effects of the surgical approach.

Studies involving multiple centers and prospective cohorts with larger sample sizes are recommended for validation purposes.

The surgery-first approach is a safe and efficient alternative to the surgery-late approach, with comparable results in condylar remodeling and surgical precision. The integration of AI tools improves the efficiency, accuracy, and scalability of CBCT analyses, which points out their potential in promoting orthognathic surgery. These results encourage the wider implementation of the SFA, especially in combination with state-of-the-art imaging technology, to optimize patient results while shortening treatment times.

## Data Availability

The data are contained within the article.
